# P-1827. Clinical Significance of Indeterminate Anti–Hepatitis C Antibody Test Results in Cancer Patients

**DOI:** 10.1093/ofid/ofaf695.1996

**Published:** 2026-01-11

**Authors:** Joanne Arvelaez Pascucci, Khalis Mustafayev, Ying Jiang, Eduardo Yepez Guevara, Harrys A Torres

**Affiliations:** The University of Texas MD Anderson Cancer Center, Houston, Texas, USA., houston, Texas; The University of Texas MD Anderson Cancer Center, Houston, Texas, USA., houston, Texas; The University of Texas MD Anderson Cancer Center, Houston, Texas; The University of Texas MD Anderson Cancer Center, Houston, Texas, Houston, TX; The University of Texas MD Anderson Cancer Center, Houston, Texas, USA., houston, Texas

## Abstract

**Background:**

Cancer patients are screened for hepatitis C virus (HCV) infection using an anti-HCV antibody (Anti-HCV) test. However, the clinical significance of an indeterminate anti-HCV result is unknown. We sought to study the clinical significance of an indeterminate anti-HCV result in cancer patients.

**Figure.**
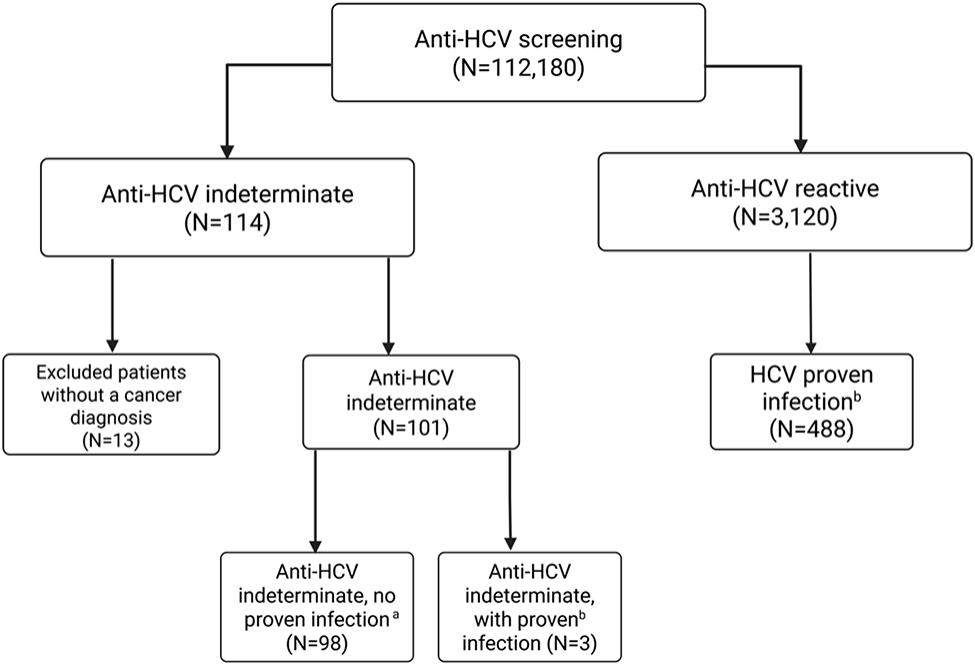
Patient flow chart Abbreviation: Anti-HCV, anti–hepatitis C antibody a Patients with undetectable HCV RNA in the absence of HCV treatment. b Patients with detectable HCV RNA or a history of HCV treatment.

**Table.**
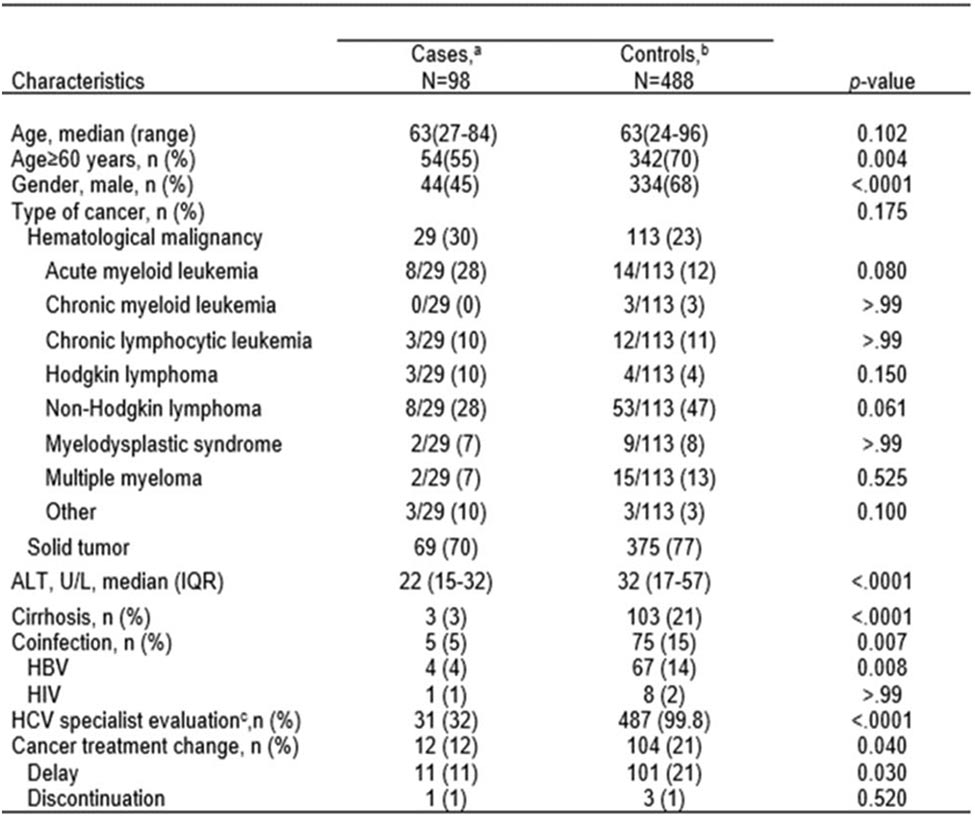
Characteristics of cases and controls. Abbreviations: ALT, alanine aminotransferase; IQR, interquartile range; HBV, hepatitis B virus; HCV, hepatitis C virus. a Patients with indeterminate anti-HCV antibody results without proven HCV infection. b Patients with reactive anti-HCV antibody results with proven HCV infection. c Evaluation by an infectious disease or hepatology specialist.

**Methods:**

We conducted a case-control study of cancer patients who were screened for HCV infection using ARCHITECT Anti-HCV (Abbot laboratories, Abbot Park, IL, USA) at our institution between October 2016 and July 2024. Patients were categorized as having indeterminate or reactive anti-HCV results. We collected information on patients’ demographics, cancer type and stage, type of anticancer therapy, co-infections (hepatitis B virus [HBV] or HIV), and management after anti-HCV testing. Cases had indeterminate anti-HCV results and undetectable HCV RNA in the absence of HCV treatment. Controls had reactive anti-HCV results and proven HCV infection (detectable HCV RNA or a history of HCV treatment). The characteristics of the cases and controls were compared using univariate and multivariate logistic regression analysis.

**Results:**

Of the 112,180 patients who underwent anti-HCV screening during the study period, 114 (0.1%) had indeterminate anti-HCV results; of these patients, 101 had cancer and were analyzed further (Figure). Anti-HCV testing was repeated in 24 (24%) patients; most had non-reactive (n=11; 46%) or indeterminate (n=9; 38%) results. Of all 101 patients with indeterminate results, 98 (97%) did not have proven HCV infection (Table), but 3 (3%; all were men older than 60 years, 2 of them with hematological malignancy and 1 with a solid tumor) had HCV infection. Multivariate analysis showed that patients who were men (adjusted odds ratio [aOR] 2.22, 95% confidence interval [CI] 1.40-3.53, p< 0.001), older than 60 years (aOR 1.61, 95% CI 1.01-2.56, p=0.046),or who had HBV or HIV coinfection (aOR 3.13, 95% CI 1.21-8.11, p=0.019), or cirrhosis (aOR 6.67, 95% CI 2.05-21.69, p=0.002) were more likely to have proven HCV (controls).

**Conclusion:**

Indeterminate anti-HCV results are uncommon in cancer patients and rarely represent HCV infection. This study provides predictors of proven HCV infection.

**Disclosures:**

Harrys A. Torres, MD, Principal investigator for research grants from the National Cancer Institute, and Gilead Sciences: Grant/Research Support

